# The Value of Predicting Human Epidermal Growth Factor Receptor 2 Status in Adenocarcinoma of the Esophagogastric Junction on CT-Based Radiomics Nomogram

**DOI:** 10.3389/fonc.2021.707686

**Published:** 2021-10-14

**Authors:** Shuxing Wang, Yiqing Chen, Han Zhang, Zhiping Liang, Jun Bu

**Affiliations:** Department of Radiology, Guangzhou Red Cross Hospital Affiliated to Jinan University, Guangdong, China

**Keywords:** adenocarcinoma of the esophagogastric junction, HER2, radiomics, nomogram, gastric cancer

## Abstract

**Purpose:**

We developed and validated a CT-based radiomics nomogram to predict *HER2* status in patients with adenocarcinoma of esophagogastric junction (AEG).

**Method:**

A total of 101 patients with *HER2*-positive (n=46) and *HER2*-negative (n=55) esophagogastric junction adenocarcinoma (AEG) were retrospectively analyzed. They were then randomly divided into a training cohort (n=70) and a verification cohort (n=31). The radiomics features were obtained from the portal phase of the CT enhanced scan. We used the least absolute shrinkage and selection operator (LASSO) logistic regression method to select the best radiomics features in the training cohort, combined them linearly, and used the radiomics signature formula to calculate the radiomics score (Rad-score) of each AEG patient. A multivariable logistic regression method was applied to develop a prediction model that incorporated the radiomics signature and independent risk predictors. The prediction performance of the nomogram was evaluated using the training and validation cohorts.

**Result:**

In the training (P<0.001) and verification groups (P<0.001), the radiomics signature combined with seven radiomics features was significantly correlated with *HER2* status. The nomogram composed of CT-reported T stage and radiomics signature showed very good predictive performance for *HER2* status. The area under the curve (AUC) of the training cohort was 0.946 (95% CI: 0.919–0.973), and that of the validation group was 0.903 (95% CI: 0.847–0.959). The calibration curve of the radiomics nomogram showed a good degree of calibration. Decision-curve analysis revealed that the radiomics nomogram was useful.

**Conclusion:**

The nomogram CT-based radiomics signature combined with CT-reported T stage can better predict the *HER2* status of AEG before surgery. It can be used as a non-invasive prediction tool for *HER2* status and is expected to guide clinical treatment decisions in clinical practice, and it can assist in the formulation of individualized treatment plans.

## Introduction

Adenocarcinoma of the esophagogastric junction (AEG) is a type of adenocarcinoma located at the junction of the distal end of the esophagus and the proximal end of the gastric cardia, independent of gastric cancer and esophageal cancer (1, 2). In recent years, the incidence of distal gastric cancer and proximal esophageal cancer has decreased, but the incidence of AEG continues to increase. The onset of AEG is hidden, and most patients are already in an advanced stage when diagnosed (2). At present, surgical resection is the only radical cure for AEG, but surgical treatment alone is not effective for the prognosis of AEG, resulting in a low postoperative overall survival rate and a high tumor recurrence rate. Therefore, surgical resection combined with perioperative comprehensive treatment is the main treatment plan for improving the overall survival rate after AEG (3). Van et al. (4) found that AEG had a worse prognosis than esophageal cancer and distal gastric cancer, and there were significant differences in biological behavior between AEG and the two cancer.

Trastuzumab is a monoclonal antibody against the human epidermal growth factor receptor-2 (*HER2*) receptor, and it is also a *HER2*-targeted drug. It can induce antibody-dependent cytotoxicity, inhibit *HER2*-mediated signal transduction, and hinder the lysis of the extracellular domain of *HER2* (5). *HER2* is currently the most well-established and widely used targeted drug gene. Tanner (6) and Yu et al. (7) found that the positive expression rate of *HER2* in gastric cancer of the esophagogastric junction was higher than that in distal gastric cancer. The positive expression of HER-2 in proximal gastric cancer was significantly higher than that in the distal gastric body and stomach. The therapeutic effects of advanced gastric cancer and gastroesophageal junction cancer treatment are related to *HER2* status. The higher the degree of *HER2* positivity, the better is the therapeutic effect (4, 8). Therefore, it is very important to determine the *HER2* gene expression status of AEG patients before surgery for targeted therapy.

The *HER2* expression status of AEG is evaluated by immunohistochemistry (IHC) or fluorescence *in situ* hybridization (FISH) of biopsy samples or postoperative pathological tissue (9), and the detection results of the two methods are similar. However, these are invasive examinations. For AEG patients, it is difficult to perform multiple tests or follow-up assessments of *HER2* status during routine diagnosis and treatment (10). At present, the research of non-invasive CT scanning methods to predict pathological or histological characteristics has gradually become a hot topic in clinical research. Conventional abdominal CT is widely used for preoperative examination of gastric cancer, but it is mostly used for the evaluation of gastric cancer staging and lymph node metastasis, and the evaluation of histology and genetic status of gastric cancer is limited (11, 12). Therefore, this study used radiomics to explore a new non-invasive inspection method to evaluate the *HER2* gene expression status of AEG.

Radiomics has been increasingly used in cancer research in recent years, especially in the analysis of tumor heterogeneity, which has unique advantages. In radio-genomics, imaging features are related to genetic features, and the greater the genomic heterogeneity of tumor tissues, the worse the prognosis (13). Radiomics is further expanded on the basis of radio-genomics, which hypothesizes that genomic heterogeneity at the microscopic level can correspond to heterogeneity within the tumor, and changes in the microenvironment within the tumor can be expressed on macroscopic images (14). Therefore, the development of radiomics provides a new approach for overcoming the limitations of traditional biopsy methods. If radiomics can be used to quantitatively analyze AEG lesions to predict *HER2* gene status, it can not only prevent invasive examinations, but can also be easily and repeatedly quantitatively analyzed. Therefore, the purpose of this study was to develop a nomogram on CT-based to predict the preoperative *HER2* status of AEG patients and to provide a nce for clinical decision-making.

## Materials and Methods

### Patients

This study retrospectively collected data from 437 patients with gastric cancer at Guangzhou Red Cross Hospital from October 2014 to January 2021. Excluding cases that did not meet the requirements, a total of 101 patients were enrolled according to the inclusion criteria (detailed below). All enrolled patients were randomly divided into training and verification cohorts at a ratio of 7:3. In the training cohort (n = 70), there were 38 *HER2*-negative cases and 32 *HER2*-positive cases, while in the verification cohort (n = 31), there were 17 *HER2*-negative cases and 14 *HER2*-positive cases.

The inclusion criteria were as follows: 1) postoperative pathologically confirmed AEG [AEG according to the diagnostic criteria of the 8th edition of the AJCC Cancer Staging Manual (15)]; 2) enhanced CT of the upper abdomen or the whole abdomen performed within one month before gastrectomy; 3) the postoperative pathological tissue underwent IHC detection to evaluate the *HER2* status; and 4) no radiotherapy or chemotherapy before surgery. The exclusion criteria were as follows:1) distant organ metastases before and during the operation; 2) incomplete clinical or pathological information; and 3) poor CT image quality, and inability to distinguish tumor lesions.

According to the American Joint Committee on Cancer (AJCC) Cancer Staging Manual 8th edition, combined with CT axial slice and multiplanar reformation (MPR) to evaluate the location of AEG (16). The staging of esophageal adenocarcinoma is based on the distance between the center of the tumor and gastric cardia ≤ 2 cm, and the staging of gastric cancer is based on a distance> 2 cm. Patient clinical and imaging data were collected, including sex, age, CT-reported TN stage, tumor carcinoembryonic antigen (CEA) level, tumor thickness, and *HER2* status.

### Clinical and Imaging Data

Studies have shown that multi-slice spiral CT (MSCT) has been widely used to assess the preoperative staging of tumors, and the accuracy of CT for T staging is 75–85%. The T staging in this study was based on previous research standards (12, 17). CT-reported T stage criteria for gastric cancer: 1) In T1, the inner layer (showing two layers of stomach wall) or the inner and middle layer (showing 3 layers of stomach wall) thickened and obviously strengthened, or it may only show obvious strengthening; 2) in T2, the tumor involves the entire thickness of the stomach wall, but the outer edge is smooth; 3) in T3, tumors involve the entire thickness of the stomach wall, with irregular or nodular protrusions on the outer edge; and 4) in T4, the entire thickness of the stomach wall is obviously strengthened, and the fat gap around the lesion disappears or the adjacent tissues and organs are invaded. T staging criteria for esophageal cancer: 1) The thickness of the esophageal wall at T1 is 3–5 mm thicker than normal; 2) compared with the normal value, the wall thickness of the esophagus at T2 is increased by more than 5 mm, but not more than 1.5 cm, the tumor is obviously enhanced, and the lumen appears slightly narrowed; 3) The thickness of the esophageal wall at T3 is thicker than normal by more than 1.5 cm, nodular protrusions are clearly visible locally, and the lumen is narrowed, but the tumor does not invade adjacent organs; 4) in T4, the tumor lesions in the esophagus have completely grown out of the esophageal wall, the lumen is severely narrowed, and the tumor has severely invaded adjacent organs. Tumor thickness: measurement of the maximum thickness of the tumor, that is, the maximum vertical distance from the surface of the lesion to the deepest infiltration. CT-reported N stage criteria: Mediastinal lymph nodes with a short diameter> 10 mm; peripheral gastric lymph nodes with a short diameter> 6 mm or peripheral extragastric lymph nodes with a short diameter> 8 mm are regarded as lymph node metastases. According to the CEA level assessment standard of the Laboratory of Guangzhou Red Cross Hospital, the normal CEA level is ≤ 6.3 ng/ml, and the abnormal CEA level is > 6.3 ng/ml. All TN stages and tumor thicknesses were determined by two radiologists with 15 years of experience in abdominal imaging diagnosis. Results that are inconsistent and final results are discussed.

### CT Image Acquisition Protocol

All patients fasted for more than 8 hours and were instructed to drink 600–1000 mL of water before the CT scan. Subsequently, a Philips Brilliance 64-slice spiral CT scanner was used to perform a contrast-enhanced scan of the patient’s abdomen, and the scan range covered the entire stomach area. Scanning parameters: The patient was in the supine position, the tube voltage was 100–120 kV, the tube current was 250 mA, and FOV 360 × 360mm. The pitch was 0.75 mm, the layer thickness was 3 mm, the spiral scanning mode was used, and the rotation time was 0.75s. After the CT, the patient used an automatic power pump syringe (Ultravist, 300 mg/ml, Schering, Germany) to apply an iodine contrast agent (1.5 mL/kg; Ultravist 370; Bayer Schering Pharma, Germany). The dose was injected into the antecubital vein, and the injection rate was 3.5 mL/s. The acquisition times of CT images in the arterial phase, portal phase, and delay phase were 30 s, 50 s, and 180 s after injection respectively.

### *HER2* Status Determination

IHC was used to evaluate the results of *HER2* status according to the gastric cancer scoring system as follows: an IHC score of 0 or 1+ indicates a *HER2*-negative status, and 3+ suggests *HER2*-positive status. Cases with an IHC score of 2+ are judged to be “indeterminate” cases, and further FISH testing is required to confirm *HER2* status. If there is gene amplification, it is judged as *HER2* positive, and if there is no gene amplification, it is judged as *HER2* negative.

### Tumor Segmentation

**Figure 1:**
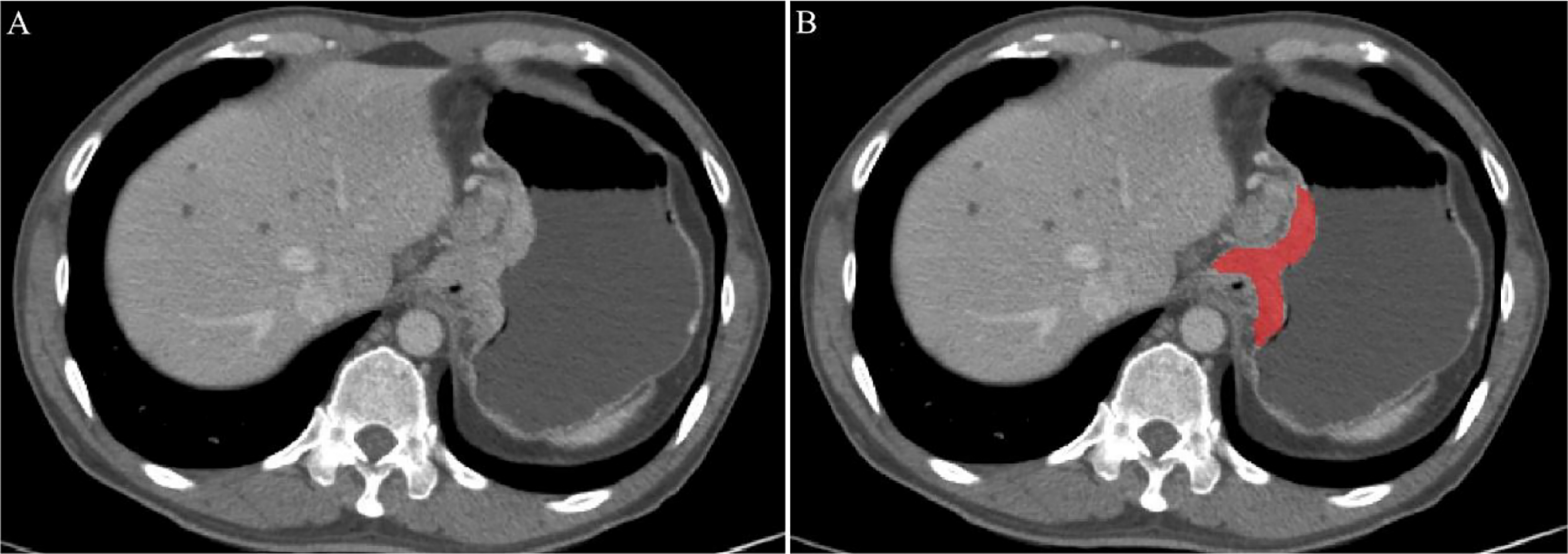
An example of manual segmentation in Adenocarcinoma of the esophagogastric junction (AEG). **(A)** Localized thick wall of esophagogastric junction with enhancement is observed on the portal venous phase computed tomography (CT) image; **(B)** Segmentation of the same axial slice.

CT images of AEG patients were retrieved from the picture archiving and communication systems (PACS) of Guangzhou Red Cross Hospital. The patient’s abdominal enhanced CT image was exported in the format of digital imaging and communications in medicine format (DICOM). The portal phase (PP) CT image was selected manually by two radiologists (observers 1 and 2). Observer 1 was a radiologist with 3 years of experience in the diagnosis of abdominal diseases, and observer 2 was a radiologist with two years of experience in the diagnosis of abdominal diseases. The ITK-SNAP (version 3.8.0, http://www.itksnap.org) image processing software was used to draw along the edge of the lesion to obtain the largest cross-sectional region of interest (ROI) of the AEG lesion, as shown in **Figure 1**. Observers 1 and 2 delineated the lesions in all patients with AEG. One week later, observer 1 delineated all the lesions again. If the segmented lesions are inconsistent between the groups and within the group, it should be judged by a radiologist with more than 15 years of experience in the diagnosis of abdominal diseases (observer 3). The final result is based on the lesions segmented by observer 3 to ensure the consistency of tumor segmentation within the observer and between the observer groups. During the delineation process, the gastric air, necrotic area, and adipose tissue in the ROI were excluded.

### Radiomics Feature Extraction and Screening

The ROI of each patient was imported into Python (version 3.7, https://www.python.org) software, the pyradiomics software package was used for radiomics feature extraction, and resampling and gray-scale normalization of the image was performed. All image data is resampled by linear interpolation, the pixel size is 0.5 mm×0.5 mm, and then the minimum and maximum normalization is used to normalize the image intensity range from 1 to 100 to obtain the same image intensity distribution.

Intraclass correlation coefficient (ICC) was used to evaluate the agreement between the intraobserver. The intraobserver ICC was calculated from the two measurement results of observer 1. ICC> 0.75 is considered to be consistent. We used the least absolute shrinkage and selection operator (LASSO) logistic regression method of five-fold cross-validation to select the best radiomics features in the training cohort, and then combined them linearly and used the radiomics signature formula to calculate the radiomics score (Rad-score) of each gastric cancer patient.

### Construction of Predictive Model

Multivariable analysis was performed to develop a prediction model by combining the Rad-score and gender, age, T stage, N stage, tumor CEA level, and tumor thickness with P values less than 0.05 in the univariable analysis. In the training cohort, to promote the clinical application value of the prediction model, we visualized the model as a radiomics nomogram based on multivariable logistic analysis.

### Radiomics Nomogram Prediction Performance

The predictive performance of the radiomics nomogram was evaluated by constructing the receiver operator characteristic (ROC) curve and calibration curve. Decision curve analysis (DCA) was performed to estimate the clinical utility of the radiomics nomogram in the training cohort.

### Statistical Analysis

IBM SPSS Statistics (Version 22.0; IBM Corp., New York, USA) and R software (version 3.4.1, http://www.R-project.org) were used for the statistical analyses. Univariate analysis was used to evaluate clinical baseline data. The continuous variables conforming to the normal distribution were statistically analyzed by $\bar{x}\pm s$ , and the t-test was used to determine whether there were statistical differences between the two groups. Non-compliance with the normal distribution is represented by the median (interquartile range), and the Mann-Whitney U test is used for comparison. Categorical variables were expressed as frequency and percentage, and the differences between the two groups were compared using the chi-square test or Fisher’s exact test. The “glmnet” package was used for least absolute shrinkage and selection operator (LASSO) logistic regression analysis. The receiver operating characteristic (ROC) plots of radiomics signature were performed with the “pROC” package. Calibration plots were done with the “rms” package. The “rmda” package was applied for decision curve analysis (DCA).

## Results

### Clinical Characteristics

Among the 101 patients with AEG, there were *HER2*-positive (n=46) and *HER2*-negative (n=55) cases. The patients were randomly divided into a training cohort of 70 cases and a verification cohort of 31 cases. In the training and validation cohorts, there were no significant differences in age, sex, or tumor CEA levels between *HER2*-positive and *HER2*-negative AEG patients (P > 0.05). In the training cohort, there were significant statistical differences in tumor thickness and CT-reported TN stage between *HER2*-positive and *HER2*-negative AEG patients (P < 0.05). In the verification cohort, there were no statistically significant differences between the CT-reported TN stage. In the training and validation cohorts, there were significant differences in the Rad-score between *HER2*-positive and *HER2*-negative AEG patients (P <0.001). The mean Rad-score of *HER2*-positive AEG patients was significantly higher than that of *HER2*-negative AEG patients in both cohorts. Additional details are provided in **Table 1**.

**Table 1:** Univariate logistic regression analysis of Clinical and radiomics features between HER2 status with AEG patients in the training and test cohort.

Charactristic	Training cohort		P	Validation cohort		P
	HER2-	HER2+		HER2-	HER2+	
Age (Y)	79.58 ± 6.08	80.41 ± 8.08	0.627	76.94 ± 6.28	78.50 ± 7.61	0.545
Sex			0.401			0.576
Male	29 (51.8)	27 (48.2)		16 (57.1)	12 (42.9)	
Famale	9 (64.3)	5 (35.7)		1 (33.3)	2 (66.7)	
tumor thickness (mm)	10.50 (10.00, 12.00)	13.0 (10.00, 18.75)	0.01	10.00 (7.00, 12.00)	11.00 (10.00, 12.65)	0.100
CT-reported T stage			0.001			0.141
T1	13 (92.9)	1 (7.1)		4 (100.0)	0(0)	
T2	9 (50.0)	9 (50.0)		4 (44.4)	5 (55.6)	
T3	9 (64.3)	5 (35.7)		5 (71.4)	2 (28.6)	
T4	7 (29.2)	17 (70.8)		4 (36.4)	7 (63.6)	
CT-reported N stage			0.02			0.153
N0-1	26 (66.7)	13 (33.3)		13 (65.0)	7 (35.0)	
N2-3	12 (38.7)	19 (61.3)		4 (39.4)	7 (63.6)	
CEA level			0.335			0.304
Normal	7 (43.8)	9 (56.3)		1 (25.0)	3 (75.0)	
Abnormal	31 (57.4)	23 (42.6)		16 (59.3)	11 (40.7)	
Rad-score	-9.24 (-11.81, -1.86)	4.15 (2.42, 5.91)	<0.001	-5.81 (-11.29, 1.78)	2.06 (0.01, 3.79)	0.020

CEA, carcinoembryonic antigen; HER2, human epidermal growth factor receptor 2; Rad-score, radiomics score; Tumor thickness, the maximum vertical distance from the surface of the lesion to the deepest infiltration.

### Radiomics Feature Selection and Radiomics Signature Building

**Figure 2:**
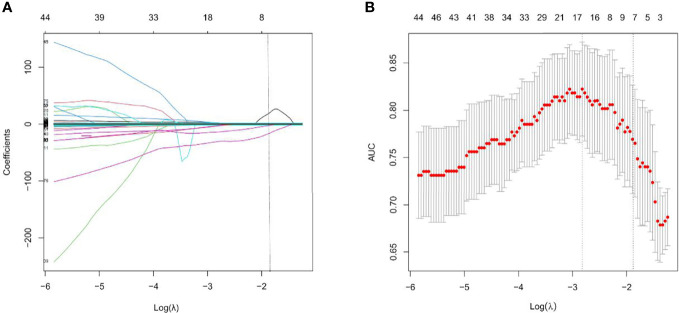
Feature selection with the least absolute shrinkage and selection operator (LASSO) binary logistic regression model. **(A)** LASSO coefficient profiles of the features. Each colored line represents corresponding coefficient of each feature. Vertical dotted line was drawn at the selected λ, where nonzero coefficients were obtained with 7 features; **(B)** Tuning parameter (λ) selection of LASSO model. The area under curve (AUC) was drawn versus log(λ). Vertical dotted line were plotted at the best value with using 5-fold cross-validation to tune parameter (λ) selection in the LASSO model.

A total of 1295 radiomics features were extracted, including 251 first-order statistics, 330 Gray Level Co-occurrence Matrix (GLCM), 224 Gray Level Size Zone (GLSZM), 224 Gray Level Run Length Matrix (GLRLM), 70 Neighbouring Gray Tone Difference Matrix (NGTDM), and 196 Gray Level Dependence Matrix (GLDM). After the consistency test and screening, 904 radiomics features with ICC> 0.75 (**Supplementary Table 1**), were retained. The optimal γ in the LASSO logistic regression analysis with 5-fold cross-validation was used to select the best radiomics feature with a non-zero coefficient, as shown in **Figure 2**. Finally, seven radiomics features were selected to construct the radiomics signature, and the Rad-score of each AEG patient was calculated. The selected radiomics features and the mathematical formula of the radiomics signature are detailed in the **Supplementary Materials**.

### Predictive Model Construction and Visualization

**Figure 3:**
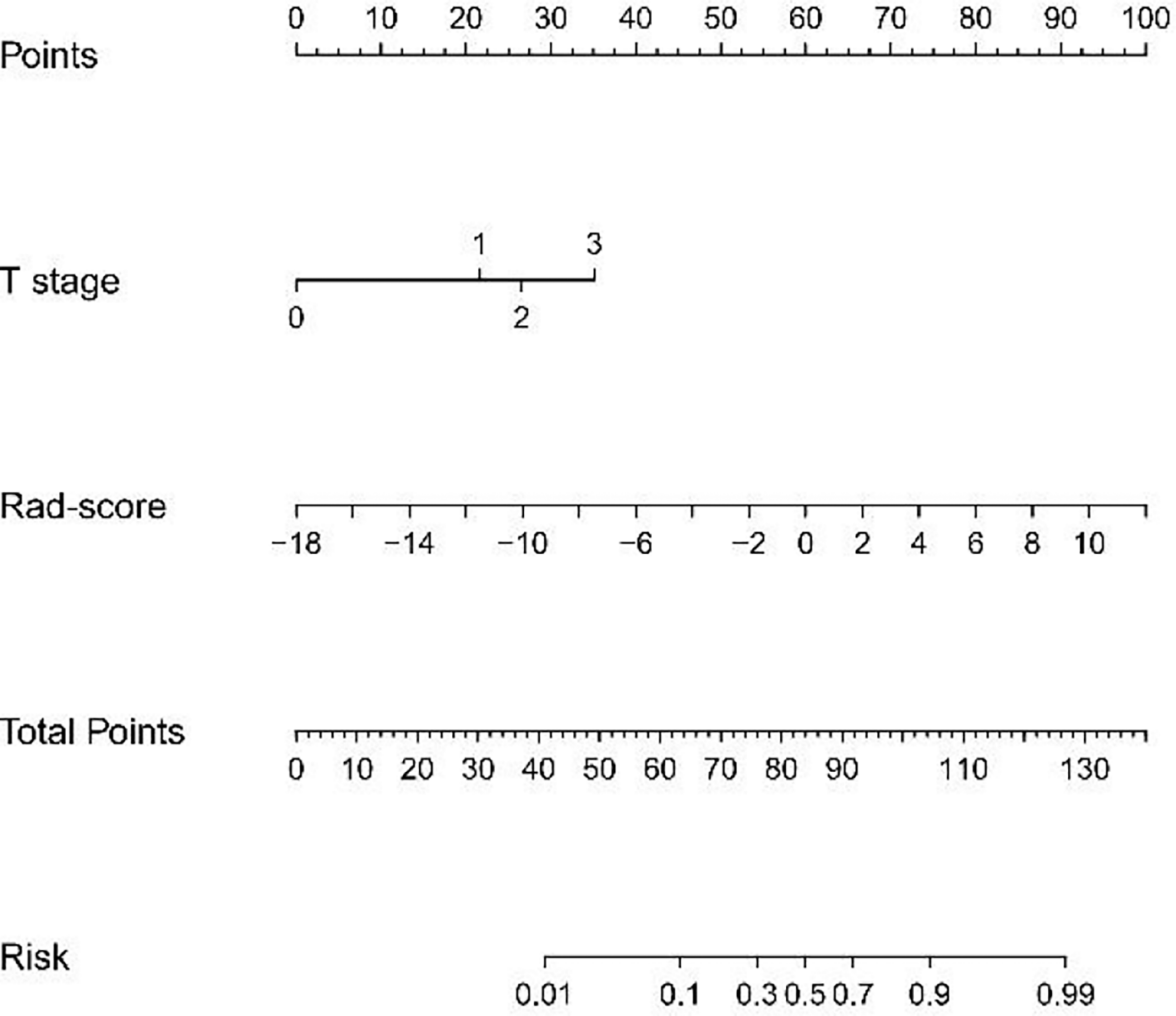
Developed radiomics nomogram. The radiomics nomogram was built in the training cohort, with the radiomics signature and the CT-reported T stage incorporated. The CT-reported T stage was considered as 0 when T0, as 2 when T2, as 3 when T3, as 4 when T4.

Multivariate logistic regression analysis of the training cohort identified CT-reported T stage and radiomics signature as independent predictors of *HER2* status in AEG patients (**Table 2**). The CT-reported T stage was integrated into the nomogram with the radiomics signature in the training cohort (**Figure 3**).

**Table 2:** Multivariate logistic regression analysis of Clinical and radiomics features between HER2 status with AEG patients in the training cohort.

Intercept and variables	β	P	OR (95% CI)
Intercept	-4.628	0.016	−
tumor thickness	0.191	0.184	1.211 (0.913, 1.605)
CT-reported T stage	1.088	0.016	2.967 (1.223, 7.201)
CT-reported N stage	1.228	0.214	3.413 (0.493, 23.612)
Rad-score	0.344	<0.001	1.411 (1.180, 1.686)

Rad-score, radiomics score; Tumor thickness, the maximum vertical distance from the surface of the lesion to the deepest infiltration.

### Predictive Performance of Radiomics Nomogram

**Figure 4:**
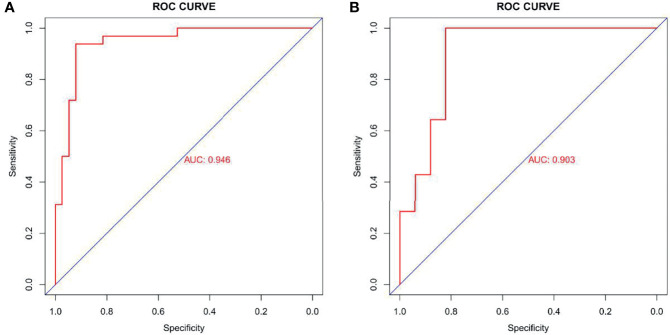
Receiver operating characteristic (ROC) curves of radiomics signature in training cohort (AUC: 0.946, 95% CI: 0.919-0.973) **(A)** and validation cohort (AUC: 0.903, 95% CI: 0.847-0.959) **(B)**. AUC, area under the curve; 95% CI, 95% confidence interval.

**Figure 5:**
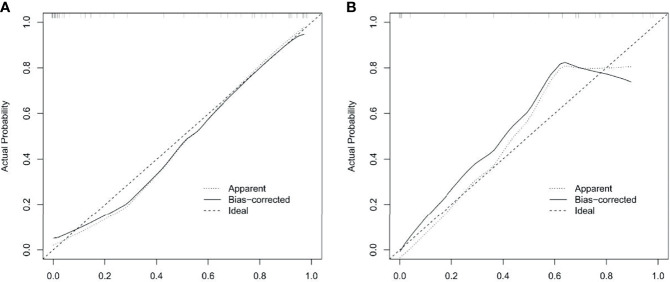
Calibration curves of radiomics nomogram in training and validation cohorts. Calibration curve plots demonstrate the calibration between predicted risks of HER2-positive status and observed outcomes of HER2-positive status in the training cohort **(A)** and the validation cohort **(B)**.

**Figure 6:**
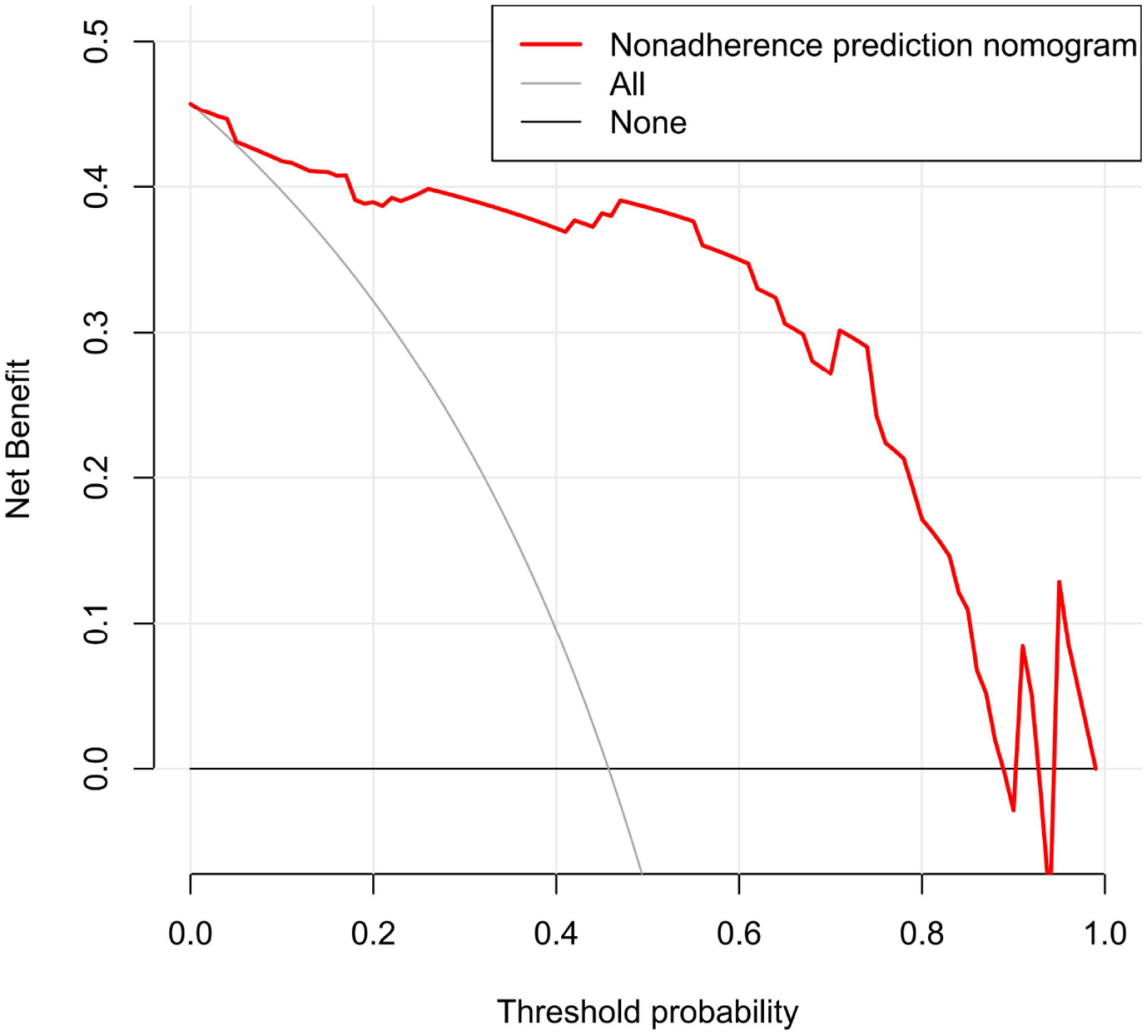
Decision curve analysis (DCA) for radiomics nomogram in training cohort. The vertical axis displays standardized net benefit. The two horizontal axes show the correspondence between risk threshold and cost: benefit ratio.

The ROC curve evaluation showed that the radiomics nomogram showed good diagnostic performance for *HER2* status. The area under the curve (AUC) of the training cohort was 0.946 (95% confidence interval [CI]: 0.919–0.973), and that of the validation cohort was 0.903 (95% CI: 0.847–0.959) (**Figure 4**). The calibration curve of the radiomics nomogram showed good agreement between the observed outcomes and predictions in both the training and validation cohorts (**Figure 5**). The DCA of the radiomics nomogram in the training cohort is shown in **Figure 6**. It showed a greater net benefit than the treat-all-patients or the treat-none schemes at a threshold probability of 10%–90%. The Hosmer-Lemeshow goodness-of-fit test (H-L) showed that the model had good calibration (*x* ^2^ = 5.496, P = 0.703).

## Discussion

In this study, we developed and validated a CT radiomics nomogram for the preoperative prediction of *HER2* status in AEG patients. The nomogram was composed of CT-reported T stage and Rad-score. It successfully stratified patients with AEG according to *HER2* status and performed well in the training and validation cohorts. At present, there are no reports on the use of radiomics to predict the *HER2* status of patients with AEG. This study explored, for the first time, both.

Traditional CT is a commonly used non-invasive examination method for the comprehensive assessment of tumor lesions and adjacent structures (18). Many previous studies have shown that the degree of AEG portal phase enhancement is an important difference between *HER2*-positive and *HER2*-negative patients (19, 20). This provides a basis for the selection of CT-enhanced images in the portal phase to delineate the lesions in this study. Portal phase-enhanced images can be used to extract more valuable radiomics features. In this study, we performed a multivariate logistic regression analysis of the clinical characteristics of the enrolled AEG patients. The results showed that CT-reported T stage was an independent risk factor for *HER2* status in AEG patients, and high *HER2* expression was significantly related to a higher T staging, which is consistent with the results of Zhang et al. (21). The reason may be related to the aggressiveness of the primary tumor. Kim et al. (22) showed that the overexpression of HER2 was positively correlated with the aggressive behavior of gastric cancer. Therefore, tumors with high HER2 expression are more likely to invade surrounding tissues, leading to higher T staging. In this study, there was no significant statistical correlation between tumor N staging and HER2 status. This is inconsistent with the research conclusion of Jørgensen et al. (23), who believe that lymph node metastasis is related to HER2 expression. Possible reasons include: 1) This study relies on the size of lymph nodes as the basis for metastasis, but CT cannot distinguish between micrometastatic lymph nodes and benign lymph nodes with inflammatory hyperplasia (24); 2) The study by Jørgensen et al. was focused on patients with advanced gastric cancer, and without conducting a separate study on the lymph node metastasis path of esophagogastric junction tumors (25). In addition, there was significant statistical correlation between tumor thickness and HER2 status, in this study. HER2 overexpression can increase the number of new blood vessels and provide more nutrients, promoting the further growth of tumor lesions, and increasing the maximum thickness of the primary tumor (26). However, the results of this study are different from the expected ones. In the multivariate analysis, there is no obvious correlation between tumor thickness and HER2 status, and it is not a predictor of HER2 status, which is possible due to the higher expression of HER2 in the esophagogastric junction is than other parts of gastric cancer (4). As the lesion grows rapidly, the central part of the lesion is prone to liquefaction and necrosis, and the solid components of the tumor are reduced. Meanwhile, the expression factors secreted by it are also reduced. Re-observation of the MSCT image may reveal a small amount of low-density shadows in some lesions, which explains the difference in results.

Accurately predicting the *HER2* status of patients with AGE has been a difficult point in recent research. The studies of Park et al. (27) showed that 18F-FDG PET/CT can use the SUVmax value to correlate with *HER2* status. Patients with *HER2*-positive gastric cancer had a higher SUVmax than did *HER2*-negative patients. However, Jinlin Song et al. (28) found in a study of the relationship between the pathological features of gastroesophageal junction cancer and 18F-FDG PET/CT, that there is no significant correlation between SUVmax and the *HER2* status of gastroesophageal junction cancer. This also proves that the related risk factors for the *HER2* status of tumors at the gastroesophageal junction are different from those of gastric cancer.

Radiomics has been increasingly used in cancer research in recent years. It is an emerging discipline that combines traditional medical image knowledge with big data analysis and precision medicine (29). Radiomics can decode tumor heterogeneity noninvasively, and there is a potential correlation between tumor genotypes and CT–based radiomics characteristics (30, 31). In the study of contrast-enhanced CT parameters of gastric adenocarcinoma, Wang et al. (32) found that radiomic features can be surrogate biomarkers for HER2 over-expression Status. Yang et al. (33) used deep radiomics features from CT images to evaluate the HER2 status of breast cancer patients. Compared to conventional handcrafted radiomics features, high-order deep radiomics features could provide further supplementary information to elevate HER2 status. The above research reports explored CT-based radiomics feature as potential biomarkers for predicting HER2 status. In recent years, integrating multiple markers into a single model has been shown to be beneficial to the individualized management of patients, and has also been shown to be superior to the use of individual marker (34–36). In this study, we examined if radiomics signature based on CT images can predict HER2 status in patients with AEG.

Radiomics can extract texture features that can be recognized by a computer from a large amount of medical image data and combined with relevant data analysis to build a specific clinical model that provides support for clinical decision-making and diagnosis. Radiomics has certain clinical application value in the prediction of tumor gene status. Previously, Li et al. (37) used a CT-based radiomics signature to predict the *HER2* status of gastric cancer, and the radiomics nomogram established by them showed good predictive ability. The AUC of the radiomics signature was 0.799 (95% CI: 0.704-0.894) in training cohort, and 0.771 (95% CI: 0.607-0.934) in validation cohort, respectively. Wang et al. (38) used random forest combined with radiomics to identify the *HER2* status of gastric cancer and used arterial phase (AP) and portal phase (PP) CT images for tumor segmentation and feature extraction, respectively. The AUC of the arterial phase radiomics model training cohort was 0.756 (95% CI: 0.656-0.840), and that of the validation cohort was 0.830 (95% CI: 0.678-0.930). The AUC of the portal phase radiomics model training cohort and validation cohort was 0.715 (95% CI: 0.612-0.804) and 0.718 (95% CI: 0.554-0.849), respectively. The above two studies based on radiomics to predict the *HER2* status of gastric cancer have shown good prediction and identification performance for the *HER2* status of gastric cancer. However, the AUC values of the training and validation cohorts of the two models were lower than the results of this study. Although the two models analyzed the location of the tumor, they did not provide a more detailed description of the tumor that occurred at the special site of the gastroesophageal junction. In this study, the nomogram CT-based radiomics signature combined with CT-reported T stage can better predict the *HER2* status of AEG before surgery. It can be used as a non-invasive prediction tool for *HER2* status and is expected to guide clinical treatment decisions in clinical practice, and it can assist in the formulation of individualized treatment plans.

This study has the following limitations: 1) It is a single-center, retrospective study with a small sample size, prone to selection bias, that a prospective randomized study with a large sample size is required to validate the findings of this study. 2) There remains some controversy regarding the CT-reported T stage of AEG patients. However, CT has become a very important part of the preoperative evaluation of AEG, especially in the evaluation of T stage, which has been widely recognized in clinical practice.

## Data Availability Statement

The raw data supporting the conclusions of this article will be made available by the authors, without undue reservation.

## Ethics Statement

The studies involving human participants were reviewed and approved by Ethical Committee of Guangzhou Red Cross Hospital. Written informed consent for participation was not required for this study in accordance with the national legislation and the institutional requirements.

## Author Contributions

SW completed the study design, data collection, statistical analysis, article writing, Radiomics analysis and chart making. YC and HZ mainly completed the data collection work. JB and ZL participated in the revision of the manuscript. All authors contributed to the article and approved the submitted version.

## Funding

This study was supported by the Science and Technology Program of Guangdong Province (2015A020210004).

## Conflict of Interest

The authors declare that the research was conducted in the absence of any commercial or financial relationships that could be construed as a potential conflict of interest.

## Publisher’s Note

All claims expressed in this article are solely those of the authors and do not necessarily represent those of their affiliated organizations, or those of the publisher, the editors and the reviewers. Any product that may be evaluated in this article, or claim that may be made by its manufacturer, is not guaranteed or endorsed by the publisher.
