# Supplementary material for: The Value of Predicting Human Epidermal Growth Factor Receptor 2 Status in Adenocarcinoma of the Esophagogastric Junction on CT-Based Radiomics Nomogram
Source: Front Oncol. 2021 Oct 14;11:707686. doi: 10.3389/fonc.2021.707686 (PMC8552039; doi:10.3389/fonc.2021.707686)
Supplement: Supplementary file 1 [file DataSheet_1.docx]

**Supplementary Materials**

**Supplementary Table 1** the data of intra-group correlation coefficient

| **Feature** | **ICC (95%CI)** |
| --- | --- |
| logarithm_glcm_Correlation | 0.96 (0.91–1) |
| wavelet.HHH_glcm_JointEntropy | 0.92 (0.88-0.96) |
| wavelet.HHL_firstorder_Kurtosis | 0.83 (0.78-0.87) |
| wavelet.LHL_firstorder_90Percentile | 0.79 (0.0.75-0.83) |
| wavelet.LLH_glcm_InverseVariance | 0.81 (0.73-0.90) |
| wavelet.LLH_glrlm_RunVariance | 0.93 (0.86-0.95) |
| wavelet.LLL_glszm_GrayLevelNonUniformityNormalized | 0.82 (0.78-0.86) |

**Radiomics score calculation formula:**

Radiomics score = -26.395 $+$ 0.041 $\times$ logarithm_glcm_Correlation $+$ 14.902 $\times$ wavelet.HHH_glcm_JointEntropy $-$ 0.223 $\times$ wavelet.HHL_firstorder_Kurtosis $+$ 0.009 $\times$ wavelet.LHL_firstorder_90Percentile $-$ 3.585 $\times$ wavelet.LLH_glcm_InverseVariance $+$ 0.574 $\times$ wavelet.LLH_glrlm_RunVariance $-$ 19.134 $\times$ wavelet.LLL_glszm_GrayLevelNonUniformityNormalized

**Radiomics Feature Extraction**

*First-order statistical features*

First-order statistics describe the distribution of voxel intensities within the image region defined by the mask through commonly used and basic metrics.

Let:

- X be a set of $N_{P}$ voxels included in the ROI
- P$\left( i \right)$ be the first order histogram with $N_{g}$ discrete intensity levels, where $N_{g}$ is the number of non-zero bins, equally spaced from 0 with a width defined in the binWidth parameter.
- $p\left( i \right)$be the normalized first order histogram and equal to $\frac{P\left( i \right)}{N_{p}}$

The following first-order statistical features were extracted:

1. **Kurtosis:**

$$\frac{\boldsymbol{\mu}_{\boldsymbol{4}}}{\boldsymbol{\sigma}_{\boldsymbol{4}}}\boldsymbol{=}\frac{\frac{\boldsymbol{1}}{\boldsymbol{N}_{\boldsymbol{p}}}\sum_{\boldsymbol{i=1}}^{\boldsymbol{N}_{\boldsymbol{p}}} \left( \boldsymbol{X}\left( \boldsymbol{i} \right)\boldsymbol{-}\bar{\boldsymbol{X}} \right)^{\boldsymbol{4}}}{\left( \frac{\boldsymbol{1}}{\boldsymbol{N}_{\boldsymbol{p}}}\sum_{\boldsymbol{i=1}}^{\boldsymbol{N}_{\boldsymbol{p}}} \left( \boldsymbol{X}\left( \boldsymbol{i} \right)\boldsymbol{-}\bar{\boldsymbol{X}} \right)^{\boldsymbol{2}} \right)^{\boldsymbol{2}}}$$

2. **90Percentile:**

**The 90^th^ percentile of X**

*Gray-Level Co-Occurrence Matrix based features (GLCM)*

GLCM based features were second-order statistical texture features, which are defined as a matrix P$\left( i,j | \delta,\theta\right)$to indicate the relative frequency with intensity values of pixels (*i* and *j*) at the distance of *δ* in direction *θ*.

Let:

- P$\left( i,j \right)$ be the co-occurence matrix for an arbitrary $\delta$ and $\theta$
- p$\left( i,j \right)$ be the normalized co-occurence matrix and equal to $\frac{P\left( i,j \right)}{\sum P\left( i,j \right)}$
- $N_{g}$ be the number of discrete intensity levels in the image
- $\sigma_{x}$be the standard deviation of $p_{x}$
- $\sigma_{y}$be the standard deviation of $p_{y}$

The following GLCM features were extracted:

1. **Correlation**

$$\frac{\sum_{\boldsymbol{i=1}}^{\boldsymbol{N}_{\boldsymbol{g}}} \sum_{\boldsymbol{j=1}}^{\boldsymbol{N}_{\boldsymbol{g}}} \boldsymbol{P}\left( \boldsymbol{i,j} \right)\boldsymbol{ij-}\boldsymbol{\mu}_{\boldsymbol{x}}\boldsymbol{\mu}_{\boldsymbol{y}}}{\boldsymbol{\sigma}_{\boldsymbol{x}}\left( \boldsymbol{i} \right)\boldsymbol{\sigma}_{\boldsymbol{y}}\left( \boldsymbol{j} \right)}$$

2. **Joint Entropy**

$$\sum_{i=1}^{N_{g}} \sum_{j=1}^{N_{g}} \left( p\left( ij \right) \right)^{2}$$

3. **Inverse Variance**

$$\sum_{\boldsymbol{k=1}}^{\boldsymbol{N}_{\boldsymbol{g}}\boldsymbol{-1}} \frac{\boldsymbol{P}_{\boldsymbol{x-y}}\left( \boldsymbol{k} \right)}{\boldsymbol{k}^{\boldsymbol{2}}}$$

*Gray Level Run Length Matrix based features (GLRLM)*

GLRLM based features were high-order statistical texture feature, which were defined as $p\left( i,j | \theta\right)$ to indicate the number of times j and gray level i appear consecutively in the direction $\theta$.

Let:

- $p\left( i,j | \theta\right)$ be the run-length matrix P for a direction $\theta$
- $N_{g}$be the number of discrete intensity values
- $N_{r}$ be the number of different run lengths

The following GLRLM features were extracted:

**Run Variance (RV)**

$\sum_{\boldsymbol{i=1}}^{\boldsymbol{N}_{\boldsymbol{g}}} \sum_{\boldsymbol{j=1}}^{\boldsymbol{N}_{\boldsymbol{r}}} \boldsymbol{p}\left( \boldsymbol{i,j} | \boldsymbol{\theta} \right)\left( \boldsymbol{j-\mu} \right)^{\boldsymbol{2}}$**,**

$$\mu=\sum_{\boldsymbol{i=1}}^{\boldsymbol{N}_{\boldsymbol{g}}} \sum_{\boldsymbol{j=1}}^{\boldsymbol{N}_{\boldsymbol{r}}} \boldsymbol{p}\left( \boldsymbol{i,j} | \boldsymbol{\theta} \right)\boldsymbol{j}$$

*Gray Level Size Zone Matrix based features (GLSZM)*

GLSZM based features were high-order statistical texture features, which were defined as *P*(*i, j*) to indicate the areas of size j and gray level i.

Let:

- P$\left( i,j \right)$ be the size zone of matrix P
- $N_{p}$ be the number of voxels in the ROI
- $N_{s}$ be the number of discreet zone sizes in the image
- $N_{z}$ be the number of zones in the ROI, which is equal to $\sum_{i=1}^{N_{g}} \sum_{j=1}^{N_{s}} P\left( i,j \right)$ and $1\leq N_{z}\leq N_{p}$

The following GLSZM features were extracted:

**Gray Level Non-Uniformity Normalized (GLN)**

$$\frac{\sum_{\boldsymbol{i=1}}^{\boldsymbol{N}_{\boldsymbol{g}}} \left( \sum_{\boldsymbol{j=1}}^{\boldsymbol{N}_{\boldsymbol{s}}} \boldsymbol{P}\left( \boldsymbol{i,j} \right) \right)^{\boldsymbol{2}}}{\boldsymbol{N}_{\boldsymbol{z}}}$$
