# Supplementary figures and images for: The Value of Predicting Human Epidermal Growth Factor Receptor 2 Status in Adenocarcinoma of the Esophagogastric Junction on CT-Based Radiomics Nomogram
Source: Front Oncol. 2021 Oct 14;11:707686. doi: 10.3389/fonc.2021.707686 (PMC8552039; doi:10.3389/fonc.2021.707686)

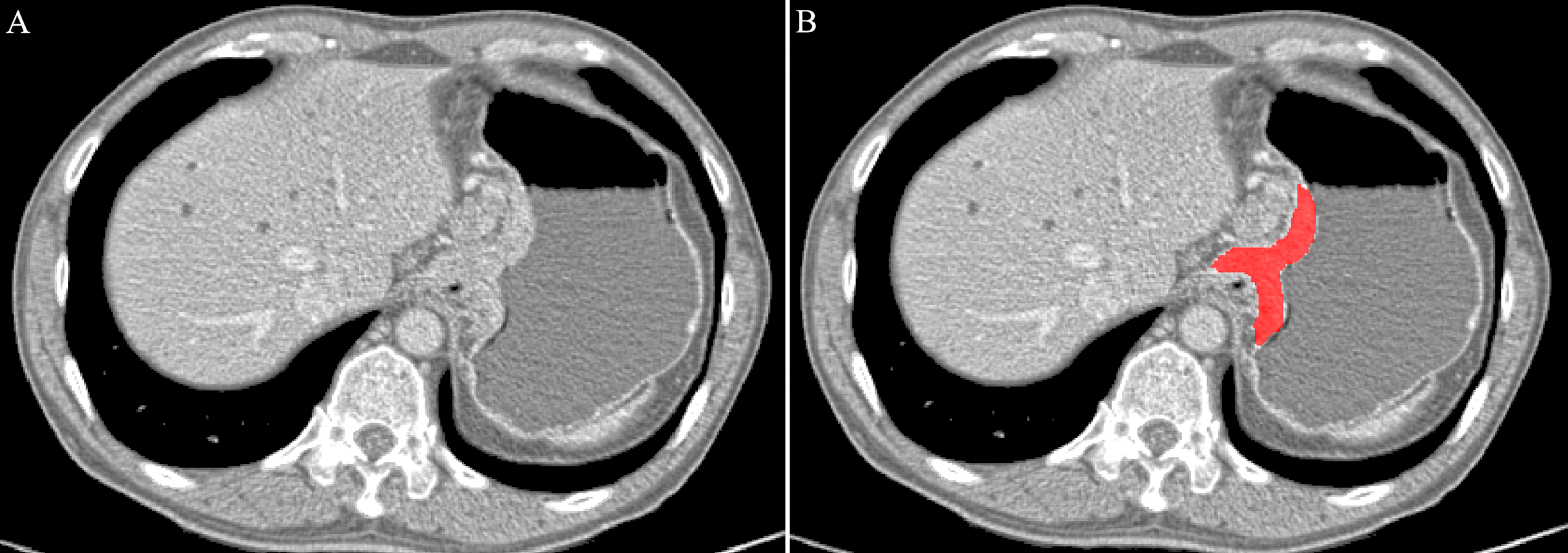

Supplement: Supplementary file 2 [file Image_1.tif]
